# Effects of Tannin Supplementation in Diet on the Resistance to Ammonia Stress of Pacific White Shrimp *Litopenaeus vannamei*

**DOI:** 10.1155/2024/5539701

**Published:** 2024-05-13

**Authors:** Xiuhong Zhang, Han Gong, Ping Chen, Jiajia Wang, Zhao Chen, Zhiqiang Chang, Jitao Li

**Affiliations:** ^1^State Key Laboratory of Mariculture Biobreeding and Sustainable Goods, Yellow Sea Fisheries Research Institute, Chinese Academy of Fishery Sciences, Qingdao 266071, China; ^2^Laboratory for Marine Fisheries Science and Food Production Processes, Qingdao Marine Science and Technology Center, Qingdao 266237, Shandong, China

## Abstract

Tannin (TA), as a natural phenolic compound with strong antioxidant activity, has been used as a feed additive for various animals. In this study, we fed a diet containing 800 mg/kg of tannin on *Litopenaeus vannamei* for 56 days and then subjected to acute ammonia stress for 48 hr to investigate the effect of dietary tannin on the ammonia stress response of *L. vannamei* through transcriptomic and metabolomic analysis. The transcriptome analysis indicated that ammonia stress-induced differential expression of 4,185 genes, while tannin-fed shrimp only had 964 differentially expressed genes. Compared with the TA_0 group, 59 pathways were significantly altered, and the pathways of “starch and sucrose metabolism,” “retinol metabolism,” “arachidonic acid metabolism,” “lysosome,” and “amino sugar and nucleotide sugar metabolism” were highly enriched in the TS_0 group. Compared with the TS_0 group, six pathways were significantly altered, and the pathways of “dilated cardiomyopathy,” “complement and coagulation cascades,” “cardiac muscle contraction,” “fructose and mannose metabolism,” “cGMP-PKG signaling pathway,” and “beta-alanine metabolism” were significantly enriched in the TS_800 group. Metabolomic analysis showed that a total of 107 differential metabolites (DMs) were identified in the TS_0 vs. TA_0 group, while 75 DMs were identified in the TS_800 vs. TS_0 group. Based on KEGG annotation, it was found that a large amount of DM was significantly enriched in amino acid metabolism pathways in the TS_0 group, including “arginine and proline metabolism,” “alanine, aspartic acid, and glutamic acid metabolism,” “*β*-Alanine metabolism and tyrosine metabolism” indicated that tannins affect the metabolism of amino acids. The integration of DEGs and DMs indicates that dietary tannins highly alter the digestion and absorption functions of proteins, as well as the biosynthesis and metabolism of amino acids. This study provides new insights into the adaptation of Pacific white shrimp to ammonia stress and the addition of tannins to feed to enhance immune function.

## 1. Introduction

As an important commercially valuable aquaculture species, the production of *Litopenaeus vannamei* in 2022 was 5.81 million tons, accounting for approximately 51.7% of all aquaculture shrimp in the world. The rapid development of the shrimp industry is mainly attributed to the application of high-density aquaculture and high-nutrient diets [1, 2]. However, the dense growth environment has further accelerated the deterioration of water quality [3]. In particular, the accumulation of organic impurities such as feces and unused feed in water resulted in a significant increase in ammonia concentration (including NH_3_ and NH_4_^+^) [4]. Obviously, ammonia is considered to be a toxic molecule that seriously affects the health of aquatic animals [5]. A great deal of research has shown that ammonia exposure can interfere with the immunity of shrimp, leading to tissue damage and even death [6, 7]. Therefore, under semi-intensive or intensive conditions, the sustainable and healthy cultivation of *L. vannamei* has received widespread attention.

As a natural phenolic compound, tannin (TA) is widely distributed in the plant kingdom and has a large number of O-hydroxyl groups, which can effectively remove oxygen-free radicals, thus exhibiting strong antioxidant activity [8]. It can also interfere with the absorption of nutrients by bacteria, viruses, parasites, etc., reduce the permeability of the host cell membrane, prevent cell membrane adsorption, and thus have an inhibitory effect on pathogens [9–11]. In recent years, tannins have been widely used as a natural substitute for antibiotics in feed in the nutrition of a large number of animals [12] to improve the gut microbiota ecosystem, enhance gut health, and thus enhance production performance [13]. In the previous studies, we investigated the effects of dietary tannin on the growth, digestion, absorption, nonspecific immunity, and resistance to ammonia stress in *L. vannamei*. The results indicate that different concentrations of dietary tannin can increase the activities of total antioxidant capacity (T-AOC), superoxide dismutase (SOD), and polyphenol oxidase. In addition, the activities of biochemical indicators such as SOD, lysozyme (LZM), and T-AOC and the expression levels of immune genes (LZM, predicted oxidase, and Cu/Zn SOD) were higher than those of the control group at 48 hr of ammonia stress. When the addition amount was 800 mg/kg, the effect was the best [14]. However, the molecular mechanism by which tannin as a feed additive to regulate the health of *L. vannamei* is still unclear and requires further exploration.

Transcriptome-based elucidation of complex molecular responses of organisms to exogenous nutrients has been widely used in crustacean research. For example, transcriptome reveals that feeding phosphorus shrimp oil to swimming crabs not only enhances the transport of substrates such as glucose, lipids, and fatty acids but also upregulates the expression level of molting-related genes, promoting immune and energy metabolism to promote the molting and growth of swimming crabs [15]; lipid sources alter the gill membrane structure and control iron balance by providing additional energy or specific fatty acids, thereby affecting the adaptation of *L. vannamei* to low salinity [16]. Metabolomics can systematically evaluate the physiological status of organisms under disease, stress, and nutritional conditions by exploring metabolic pathways such as sugar generation, amino acid, and lipid metabolism [17, 18]. In addition, omics techniques have also been used to elucidate the molecular regulatory mechanisms of aquatic organisms under ammonia stress [19, 20]. Our preliminary research found that TA has a good effect on improving the antiammonia nitrogen stress ability of *L. vannamei*, but the underlying molecular mechanism was still unclear. Therefore, the molecular regulation mechanism of adding tannin to shrimp feed to resist ammonia stress needs to be explored in depth using integrative omics techniques.

Therefore, this study combined the advantages of transcriptome and metabolome to reveal the molecular regulatory mechanisms of dietary tannin on the ammonia stress response of *L. vannamei*. This study will help to gradually understand the advantages of tannin as a feed additive and provide molecular support for evaluating the health of *L. vannamei* and responding to ammonia stress.

## 2. Materials and Methods

### 2.1. Experimental Diet Preparation

The experimental diets were developed based on the NRC (2011) guidelines and the nutritional requirements of *L. vannamei*. The basal diet provided by Hengxing Group Co. Ltd. (Guangdong, China) was used as the control diet (TA_0). TA is a polyphenolic compound commonly found in nature with strong water solubility. The TA used in this study is a hydrolyzed tannin extracted from gallnuts, provided by Qingdao RBT Biotechnology Co., Ltd. (Qingdao, China). After crushed, sieved, and soaked, the soaked water was clarified and preheated and then spray dried, and the dry powder obtained was screened to obtain the final product. TA is completely dissolved in water before being added to the diet, mixed thoroughly with the base diet at a dose of 800 mg/kg, compressed into 2 mm particles, and stored at −20°C for later use (Table 1).

Each kilogram of composite premix contains vitamin A, 500,00 0 IU; vitamin D, 310,000 IU; vitamin E, 4,000 mg; vitamin K, 31,000 mg; vitamin B1, 500 mg; vitamin B2, 1,000 mg; vitamin B6, 1,000 mg; vitamin B12, 2 mg; niacin, 4,000 mg; calcium pantothenate, 2,000 mg; folic acid, 100 mg; biotin, 10 mg; vitamin C, 15,000 mg; iron, 10,000 mg; copper, 300 mg; zinc, 5,000 mg; manganese, 1,200 mg; iodine, 80 mg; selenium, 30 mg; cobalt, 20 mg.

### 2.2. Shrimp, Culture Conditions, and Feeding Test

Randomly select healthy *L. vannamei* with an average weight (initial weight = 0.3 ± 0.03 g) from the aquaculture ponds of Haifeng Aquaculture Co., Ltd. (Changyi, China). After 7 days of acclimatization, healthy shrimps in the mountainous stage were randomly allocated into two groups: the not adding TA groups (TA_0) and the TA addition group (TA_800). Each group included three duplicate tanks. The shrimp were raised in boxes of 500, each containing 5,000 L of inflatable seawater. The experimental shrimp were fed three times a day at 6:00, 12:00, and 18:00, with an apparent full stomach. The feeding trial lasted for 56 days, and the water quality parameters were dissolved oxygen >6 mg/L; salinity 32 ± 0.5; temperature 28 ± 0.5°C and pH 7.8–8.2. About 30% of the water in each water tank was replaced every day, and the shrimp feces and uneaten feed were cleaned out each time.

### 2.3. Ammonia Stress Experiment

After the 56-day feeding experiment was completed, 50 shrimps were randomly selected from the TA_0 group and the TA_800 group for an ammonia stress test. Based on the LC_50_ of *L. vannamei* under ammonia stress for 48 hr, this study set the ammonia concentration to 30.0 mg/L [21]. The NH_4_Cl solution was added to the seawater to keep the ammonia concentration at 30.0 mg/L and adjusted in a timely manner in order to insure stability. The ammonia concentration in seawater was measured using indophenol blue spectrophotometry (GB 17378.4-2007). The whole ammonia stress experiment lasted for 48 hr. After the experiment, calculate the survival rates of TS_0 and TS_800 groups of shrimps after 48 hr of ammonia stress (TS_0 : 15.67% and TS_800 : 30.67%). Treat the experimental tail water according to Gao et al. [22].

### 2.4. Sampling

After 48 hr ammonia stress experiment, the hemolymph of the shrimp in each tank was sampled, respectively. The groups included the control group (TA_0), the control group after ammonia stress (TS_0), the TA addition group (TA_800), and the TA addition group after ammonia stress (TS_800). Each group consisted of three replicate tanks. In detail, nine shrimps were taken from each replicate tank, and the hemolymph samples of every three shrimps were mixed into one sample, with one sample used for transcriptome analysis and two samples used for metabolomics analysis. In the end, each group had three transcriptome samples and six metabolome samples. All the samples were collected and quickly frozen in liquid nitrogen for facilitate subsequent experiments.

### 2.5. Hemocyte Transcriptomic Analysis

According to the instructions, extract total RNA from hemocyte samples and then perform gDNA removal, RNA purification, and quality testing. A sequencing library was constructed using high-quality RNA samples on the Illumina platform. After removing low-quality readings (quality score < 30) and readings containing linker sequences, the data were assembled using Trinity software for further analysis. Finally, annotate the reading with the genome of *L. vannamei* (https://www.ncbi.nlm.nih.gov/genome/?term=*Penaeus vannamei*). The supplementary materials provide a detailed experimental method.

In order to identify differential expression genes (DEGs) between different treatments of TS_0 vs. TA_0 and TS_800 vs. TS_0, the expression level of each transcript was calculated. Furthermore, functional enrichment analyses, including GO and KEGG, were conducted to determine the significant enrichment of DEGs in GO terminology and metabolic pathways. Finally, qPCR analysis of DEGs was used to validate transcriptome quality (*Supplementary 1*).

### 2.6. Hemolymph Metabolomics Analysis

After pretreatment of the samples, a total of 24 hemolymph samples from four groups were used for metabolomics analysis (*n* = 6). All hemolymph samples were obtained from the above liquid nitrogen-preserved samples and thawed 4°C. The metabolite extract was dissolved in an 80% methanol solution. Twenty microlitres of each sample were taken as a quality control sample, and the remaining samples were used for LC–MS detection. The database was used to annotate the metabolomics data, and orthogonal partial least squares discriminant analysis (OPLS-DA) was performed. The differential metabolites (DMs) of TS_ 0 vs. TA_ 0 and TS_ 800 vs. TS_ 0 were identified. Then, the pathway was analyzed for DMs and further research was conducted on biomarkers of metabolites recognized.

### 2.7. Integration Analysis of Transcriptome and Metabolome

A comprehensive analysis of DEGs and DMs revealed pathways of significant changes. First, the change pathways of DMs were screened, and then the change pathways of DEGs were annotated and identified. Then, R (v3.3.2) software was used to integrate and map the pathways of DEGs and DMs. *P* < 0.05 was considered statistically significant.

The supplementary materials provide a detailed experimental method (*Supplementary 2*).

## 3. Results

### 3.1. Hemocyte Transcription Pattern Alterations Analysis

#### 3.1.1. Identification of the DEGs

**Figure 1:**
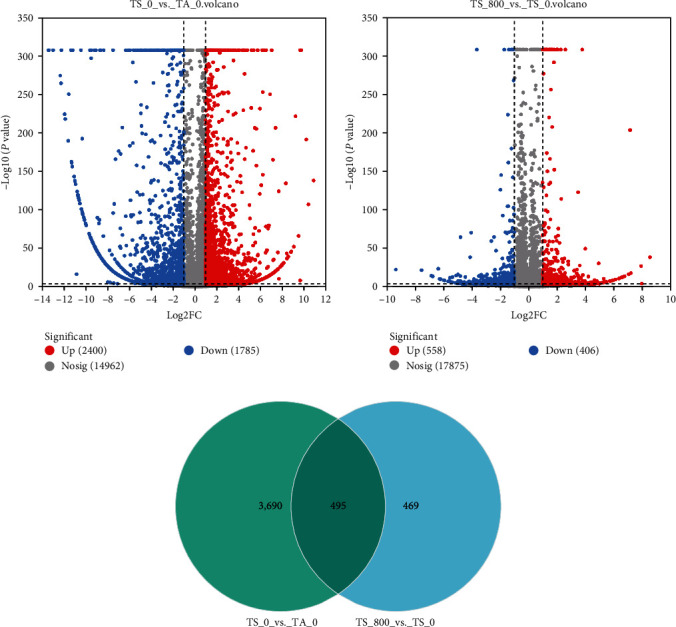
The number of differentially expressed genes between different groups: (a) volcano plot of DEGs from the transcriptomes of the TS_0 vs. TA_0 group; (b) volcano plot of DEGs from the transcriptomes of the TS_800 vs. TS_0 group; (c) Venn analysis of differential genes in the two stress groups. Significantly upregulated and downregulated are indicated in red and blue dots, respectively, and not significantly different are in gray dots.

A total of 4,185 DEGs were identified in the TS_0 vs. TA_0 group, including 2,400 upregulated and 1,785 downregulated DEGs (Figure 1(a)), and a total of 964 DEGs were identified in the TS_800 vs. TS_0 group, including 558 upregulated and 406 downregulated genes (Figure 1(b)). Among them, a total of 495 DEGs were all altered in the two comparison groups; 3,690 and 469 genes were differentially expressed in the TS_0 group and TS_800 group, respectively (Figure 1(c)).

#### 3.1.2. Functional Enrichment Analysis of DEGs

**Figure 2:**
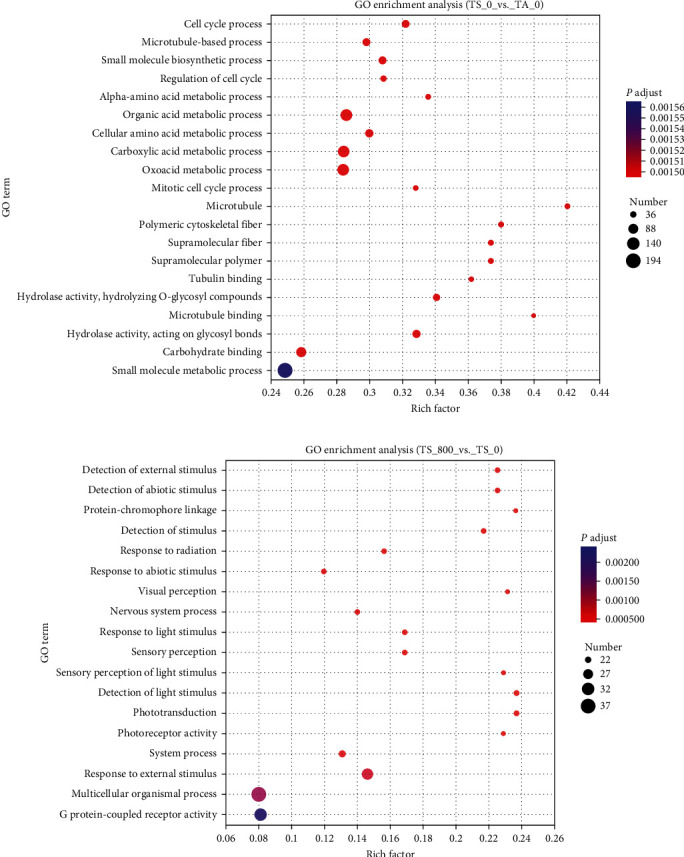
The GO enrichment analysis in the hemocyte of *L. vannamei*: (a) the GO enrichment analysis in the TS_0 vs. TA_0 group; (b) the GO enrichment analysis in the TS_800 vs. TS_0 group.

**Figure 3:**
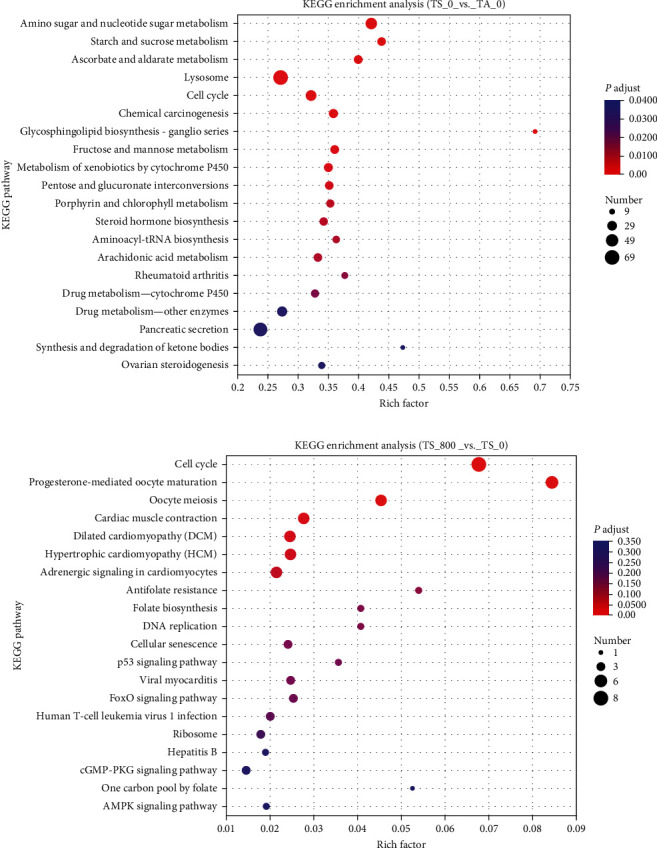
The KEGG enrichment analysis in the hemocyte of *L. vannamei*: (a) the KEGG enrichment analysis in the TS_0 vs. TA_0 group; (b) the KEGG enrichment analysis in the TS_800 vs. TS_0 group.

The DEGs were annotated by GO enrichment analysis. Compared with the TA_0 group, the metabolic-related functions were highly enriched in the TS_0 group, such as the alpha amino acid metabolic process, organic acid metabolic process, carboxylic acid metabolic process, and oxoacid metabolic process (Figure 2(a)). Compared with the TS_0 group, the functions of the perception of stimuli were highly enriched in the TS_800 group, including detection of external stimulus, detection of abiotic stimulus, etc. (Figure 2(b)). The KEGG pathway of the DEGs was further explored. Compared with the TA_0 group, 59 pathways were significantly altered, and the pathways of “starch and sucrose metabolism,” “retinol metabolism,” “arachidonic acid metabolism,” “ascorbate and aldarate metabolism,” “lysosome,” “apoptosis,” “pantothenic acid and CoA biosynthesis,” and “amino sugar and nucleotide sugar metabolism” were highly enriched in the TS_0 group (Figure 3(a)). Compared with the TS_0 group, six pathways were significantly altered, and the pathways of “dilated cardiomyopathy (DCM),” “complement and coagulation cascades,” “cardiac muscle contraction,” “fructose and mannose metabolism,” “cGMP-PKG signaling pathway,” and “beta-alanine metabolism” were significantly enriched in the TS_800 group (Figure 3(b)).

#### 3.1.3. The Characteristics of Immune-related Genes

Several DEGs were identified in the transcriptome data, which can better understand the improvement of dietary tannins on ammonia stress response in *L. vannamei* (Table 2). Under ammonia stress, many immune-related genes, such as diacylglycerol kinase, phenoloxidase activating enzyme, nuclear receptor subfamily 4, and lysozyme, were downregulated, while phenoloxidase, apoptosis-inducing factor 3, and serine protein were upregulated in the TS_0_vs._TA_0 group. However, after dietary tannin, the expression of the above genes had no significant changes in the TS_800_vs._TS_0 group.

In addition, heat shock protein (HSP) and lysozyme C were downregulated in the TS_0_vs._TA_0 group but upregulated in the TS_800_vs._TS_0 group; phenoloxidase-activating factor 3 was upregulated in the TS_0_vs._TA_0 group, but downregulated in the TS_800_vs._TS_0 group; gamma-glutamyl hydrolase was upregulated both in the TS_0_vs._TA_0 and TS_800_vs._TS_0 groups; choline transporter-like protein 1 was upregulated in the TS_800_vs._TS_0 group, but was no significant change in the TS_0_vs._TA_0 group.

### 3.2. Hemolymph Metabolome Alterations

#### 3.2.1. Identification of the DMs

**Figure 4:**
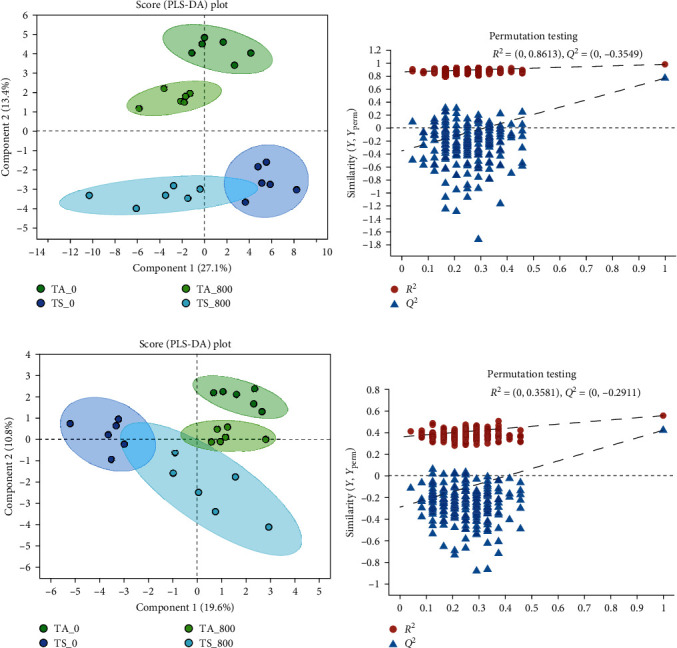
Derived PLS-DA score plots and corresponding permutation testing of PLS-DA from the metabolite profiles in the hemolymph of *L. vannamei*: (a) PLS-DA score plot of positive ions; (b) permutation testing of positive ions; (c) PLS-DA score plot of negative ions; (d) permutation testing of negative ions.

Untargeted metabolomic analysis was investigated to explore the effect of dietary tannin on the metabolic variations of the shrimp induced by ammonia stress. The PLS-DA score plots showed very significant discriminations in the two compared groups, indicating that dietary tannin altered the hemolymph metabolomics profiles of *L. vannamei* under ammonia stress (Figure 4).

#### 3.2.2. The Variation Characteristics of the Metabolites

**Figure 5:**
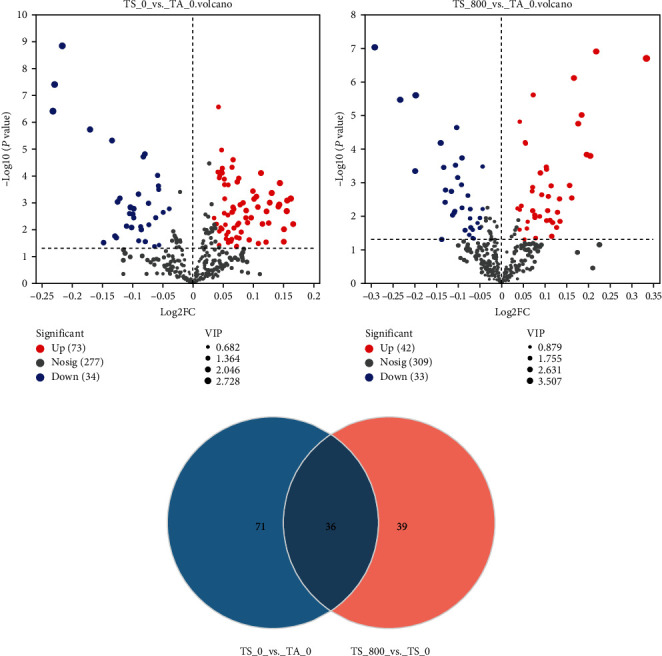
The number of the DMs between different groups: (a) volcano plot of DMs of the TS_0 vs. TA_0 group; (b) volcano plot of DMs of the TS_800 vs. TS_0 group; (c) Venn analysis of the DMs in the two comparison groups. Significantly upregulated and downregulated were indicated in red and blue dots, respectively, and not significantly different are in gray dots.

A total of 107 DMs were identified in the TS_0 vs. TA_0 group, including 34 upregulated and 73 downregulated metabolites (Figure 5(a)). A total of 75 DMs were identified in the TS_800 vs. TS_0 group, including 42 upregulated and 33 downregulated metabolites (Figure 5(b)). Among them, a total of 36 metabolites were all altered in the two comparison groups; 71 and 39 metabolites were differentially altered in the TS_0 group and TS_800 group, respectively (Figure 5(c)).

**Figure 6:**
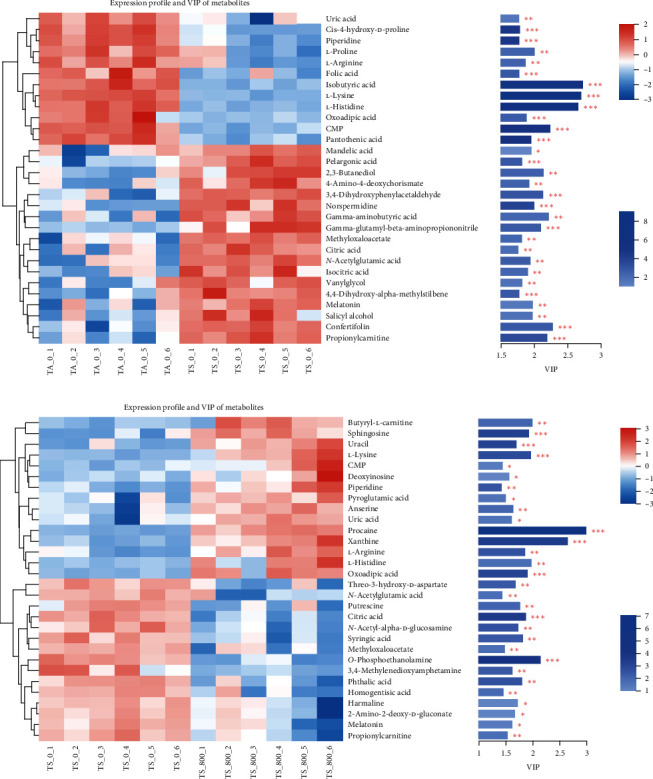
The expression profiles and VIP value of the DMs in the hemolymph of *L. vannamei*: (a) the TS_0 vs. TA_0 group; (b) the TS_800 vs. TS_0 group.

The metabolites related to stress response were identified (Figures 6(a) and 6(b)). Eleven DMs, including three amino acids (L-arginine, L-histidine, L-lysine), two organic acids (citric acid, oxoadipic acid), and other metabolites, were all identified in the TS_0 vs. TA_0 and TS_800 vs. TS_0 groups. Additionally, L-proline, two cofactors, and vitamins (folic acid and pantothenic acid) were only changed in the TS_ 800 vs. TS_ 0 group.

#### 3.2.3. Functional Annotation of the DMs

**Figure 7:**
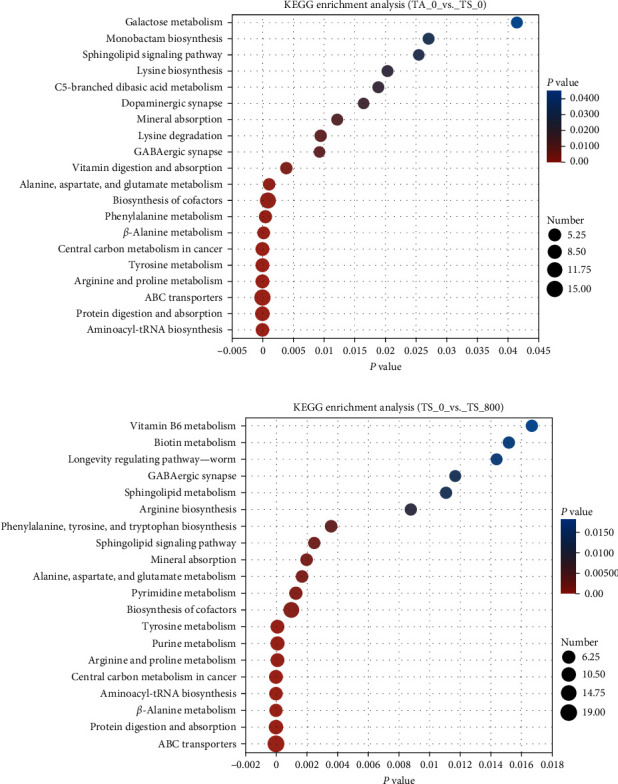
KEGG enrichment analysis in the hemolymph of *L. vannamei*: (a) the TS_0 vs. TA_0 group; (b) the TS_800 vs. TS_0 group.

Based on KEGG annotation, the DMs in different comparisons were enriched in many pathways (Figures 7(a) and 7(b)). The Figure 7 lists the top 20 significantly enriched pathways. Thereinto, the pathways of “ABC transporters,” “alanine, aspartate and glutamate metabolism,” “aminoacyl-tRNA biosynthesis,” “arginine and proline metabolism,” “beta-alanine metabolism,” “biosynthesis of cofactors,” “GABAergic synapse,” “mineral absorption,” “protein digestion and absorption,” “sphingolipid signaling pathway,” and “tyrosine metabolism” were all dominated in the TS_0_vs._TA_0 and TS_800_vs._TS_0 groups. In addition, the pathways of “arginine biosynthesis,” “pyrimidine metabolism,” “purine metabolism,” “phenylalanine, tyrosine and tryptophan biosynthesis,” “vitamin B6 metabolism,” and “longevity regulating pathway” were highly impacted in the TS_800 group.

#### 3.2.4. Integrative Analysis of Transcriptomics and Metabolomics

**Figure 8:**
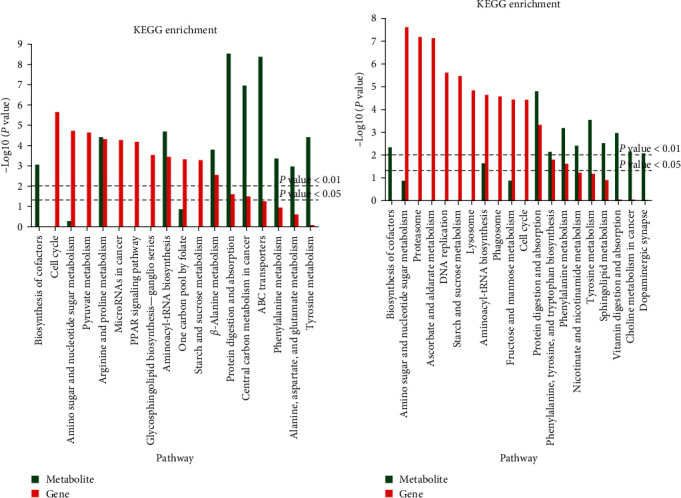
The pathways of differential genes and metabolites based on the integration analysis of transcriptomics and metabolomics: (a) the TS_0 vs. TA_0 group; (b) the TS_800 vs. TS_0 group.

According to the integration of the differential genes and metabolites, the pathways of “protein digestion and absorption” and “aminoacyl-tRNA biosynthesis” were all significantly affected in the TS_0_vs._TA_0 and TS_800_vs._TS_0 groups. Additionally, the pathways of “arginine and proline metabolism” and “beta-alanine metabolism” were significantly affected in the TS_0 vs. TA_0 group (Figure 8 (a)); the pathways of “phenylalanine, tyrosine and tryptophan biosynthesis,” and “phenylalanine metabolism” were significantly affected in the TS_800 vs. TS_0 group (Figure 8(b)).

## 4. Discussion

Current research has shown that dietary tannin can improve the health status of shrimp. As a feed additive for aquatic animals, tannin is widely used, such as *L. vannamei*, pearl gentian grouper [23], *Lateolabrax maculatus* [24], and other economically valuable aquatic animals. However, the specific molecular regulatory mechanism is not yet known. In this study, for the first time, the optimal dosage of tannin was fed to *L. vannamei*, and the advantages of transcriptome and metabolome were integrated to explore the molecular regulatory mechanism of dietary tannin on the ammonia stress response of *L. vannamei*.

### 4.1. Transcriptome Revealed that Tannin Enhanced Shrimp Resistance

Tannin has strong antioxidant activity, and numerous studies have indicated that it plays an essential role in enhancing immune function, preventing lipid peroxidation, improving intestinal microbiota, and promoting nutrient digestion and absorption [25, 26]. In this study, ammonia stress-induced differential expression of 4,185 genes and significant changes in 59 pathways, while tannin-fed shrimp only had 964 differentially expressed genes and 6 pathways significant changes were observed in the TS_800 groups. This phenomenon indicates that tannin helps maintain the homeostasis of gene transcription in shrimp.

Numerous studies have found that ammonia stress led to differential expression of immune-related genes in cephalopods, such as peptidoglycan recognition protein [27], TNF receptor-related factor 2 [28], and interleukin-17 like protein [29], indicating that ammonia stress may induce immune system disorders. In addition, similar downregulation of immune-related genes has also been observed in crustaceans [30, 31]. Crustaceans lack specific immune responses and can only depend on the innate immune system to resist complex and ever-changing pathogens and environmental conditions [32, 33]. Therefore, the activity and total protein content of immune enzymes such as alanine aminotransferase (ALT), phenol oxidase (PO), aspartate aminotransferase (AST), and lysozyme (LZM) in the hemolymph are important indicators for measuring shrimp immunity [34]. In this study, genes related to the phenolic oxidase system, such as PO and serine protease inhibitors, were identified in TS_0 group differential expression downregulation. Phenol oxidase (PO) is a copper-containing oxidase that is widely present in plants and animals. As an important part of the pro phenol oxidase activation system, PO is an important immune factor that participates in various immune responses and plays an important role in the nonspecific immune mechanism of Invertebrate [35, 36]. In the immune defense system of crustaceans, the body damage caused by the outside world, or a small amount of foreign matter, will activate the enzyme oxidase source activation system and then eliminate or resist external interference through the secretion, encapsulation, phagocytosis, and melanization of cytotoxic [32, 37, 38]. Therefore, the downregulation of differential expression of PO and serine protease inhibitors indicates that ammonia stress may lead to a decrease in shrimp immune function. It is worth noting that PO was not significant in TS_ 800 vs. TS_ 0 group, indicating that tannin addition can maintain the stability of the phenolic oxidase system, thereby maintaining the physiological homeostasis of shrimp.

In addition, HSPs, as molecular chaperones, play a crucial role in maintaining cellular protein stability by regulating protein folding, refolding, translocation, and degradation [39, 40]. In recent years, the potential role of HSPs in immunity has been recognized, especially these proteins trigger various immune responses to infection and regulate inflammation [40–42]. The results showed that heat stress could induce the expression of HSP70 at gene and protein levels, and the relationship between the upregulation of HSP70 and the activation of proPO system was found [43, 44], Phuoc et al.[45] found that, after feeding with tannin, the HSP of *L. vannamei* increased significantly in response to ammonia stress [45], which is consistent with our research results. Therefore, it is speculated that adding tannin can promote differential expression of HSPs, thereby activating the proPO system in response to ammonia stress.

In addition, other immune-related DEGs, including alkaline lysozyme, early growth response genes, diacylglycerol kinase, and retinol dehydrogenase 11, further confirmed that tannin can enhance the immune ability of shrimp. Lysozyme (LZM) can catalyze the hydrolysis of bacterial cell walls, leading to bacterial dissolution and death [46–49]. It is one of the important nonspecific immune factors in shrimp, playing an important role in resisting bacterial invasion and maintaining immune defense [50, 51]. In this study, lysozyme significantly decreased after ammonia stress but increased significantly after feeding tannin under ammonia stress. This seems to indicate that supplementing tannin in the diet can regulate the participation of lysosomes in shrimp response to ammonia stress.

From the signal pathways enriched by RNA-Seq results, it can be seen that the genes involved in the immune response of *L. vannamei* mostly complete their biological functions by regulating the upstream and downstream signaling pathways of lysosomal signaling and pancreatic secretion. Therefore, tannin may mainly reduce the inflammatory response of *L. vannamei* through the lysosomal pathway, thereby improving its immune ability.

### 4.2. Tannin-Induced Metabolic Function Changes

Based on KEGG annotations, it was found that in TS_0 groups, A lots of DMs were significantly enriched in amino acid metabolism pathways, including “arginine and proline metabolism,” “alanine, aspartate, and glutamate metabolism,” “beta-alanine metabolism,” and “tyrosine metabolism” indicated that tannin affects the metabolism of amino acids.

Amino acids, especially essential amino acids, are one of the important nutrients in shrimp and exert important effects on the normal growth and development of shrimp. L-Histidine can be converted into histamine under the action of histidine decarboxylase. Histamine is an important neurotransmitter widely present in animal tissues and is involved in regulating processes such as sleep, anxiety, hormone secretion, body temperature changes, appetite, and memory formation [52]. Research has found that L-histidine and histamine levels play an essential role in the anti-stress response process [53]. In this study, L-histidine was increased in the TS_0 group by comparing with the TA_0 group, and interestingly, L-histidine was increased in the TS_800 group by comparing with the TS_0 group, suggests that adding tannins to the diet can induce more L-histidine to maintain physiological homeostasis and resist stress response processes.

Another metabolically different amino acid is arginine. Arginine, as a functional amino acid, can enhance the immunity of aquatic animals [54, 55]. Arginine is widely involved in body metabolism through the catalysis of arginase, nitric oxide synthase, arginine glycine transferase, and arginine decarboxylase [53], such as protein synthesis, ornithine production, creatine, and dopamine synthesis, as well as the metabolism of glutamate and proline [56, 57]. Research has found that arginine plays an important role in the growth and development, immune stress, and other aspects of crayfish [58, 59]. In this research, similar changes in L-arginine and L-histidine. Therefore, adding tannin to the diet can enhance the immune ability of shrimp by regulating amino acid metabolism.

In addition, an important KEGG pathway, namely ABC transporter protein, has been discovered. As a membrane-binding protein, ABC transporters are involved in maintaining cellular osmotic pressure, bacterial immunity, and antigen presentation, which are associated with immune deficiency and cancer [60–62]. According to transcriptomic analysis, TS_0 vs. TA_0 and TS_800 vs. TS_0 showed 15 and 19 differential metabolic enrichment of ABC transport, indicated that tannin affects the transmembrane transport of substances.

Finally, the integration analysis of DMs and DEGs was combined; in TS_800 vs. TS_0, DMs and DEGs are significantly enriched in aminoacyl tRNA biosynthesis, protein digestion, and absorption, arginine and proline metabolism, cell apoptosis, glycerol phospholipid metabolism, phenylalanine, tyrosine and tryptophan biosynthesis, phenylalanine metabolism, and bile secretion. Proteins were converted into amino acids before they can be absorbed and utilized by the organism. Amino acid metabolism is also an important component of the TCA cycle [63], and aminoacyl tRNA is responsible for transporting amino acids to ribosomes for protein synthesis [54, 64]. The synthesis and metabolism of various amino acids in living organisms maintain their homeostasis. Glycerol phospholipid was the most abundant type of phospholipids in the body; in addition to forming biofilms, they were also one of the components of bile and membrane surfactants and participate in the recognition and signal transduction of proteins by cell membranes [65]. This further confirms our previous inference that tannins affect shrimp's immune function, amino acid metabolism, protein synthesis, transmembrane transport, and other processes by regulating these pathways to maintain a stable state.

## 5. Conclusions

In this study, we combined transcriptomics and metabolomics analysis for the first time to describe how the addition of tannins to the diet improves the health of *L. vannamei* and its response to ammonia stress. Tannin can enhance the nonspecific immunity of shrimp by regulating the differential expression of genes such as phenoloxidase and lysozyme, as well as corresponding signaling pathways. Besides that, a large number of DMs, including essential amino acids such as L-lysine, L-arginine, and L-histidine, confirm that tannin leads to the remodeling of shrimp metabolic function. Integration analysis showed that tannins affect protein digestion and absorption, as well as amino acid biosynthesis and metabolism. We conclude that tannin can maintain the response of *L. vannamei* to ammonia stress by affecting processes such as immunity, amino acid metabolism, protein synthesis, and transmembrane transport.

## Figures and Tables

**Table 1:** The composition of the *L. vannamei* experimental diets.

Ingredient (g/kg)	TA_0	TA_800
Fish meal	230	230
Solvent-extracted soybean meal	300	300
Whole wheat	100	100
Corn gluten (60% protein)	202	202
Dicalcium phosphate	50	50
Fish oil	33	33
Bentonite	75	75
Lecithin	1	1
Vitamin premix^1^	1	1
Mineral premix	1	1
Stable C	1	1
Lysine hydrochloride	6	6
Copper sulfate	1	1
Tannin	—	0.8

^1^The premix (multidimensional and multimineral) is provided by Qingdao Master Biotechnology Co., Ltd.

**Table 2:** Immune-related differential gene statistics.

Gene name	Gene description	TS_0_vs._TA_0	TS_800_vs._TS_0
LOC113806309	GTP-binding protein 2	Up	/
LOC113818855	GTPase-activating protein	Up	/
LOC113813424	Beta-hexosaminidase	Up	/
LOC113829328	Gamma-glutamyl hydrolase	Up	Up
LOC113826948	Choline transporter-like protein 1	/	Up
LOC113824028	Diacylglycerol kinase theta	Down	/
LOC113826282	Diacylglycerol kinase kappa	Down	/
LOC113827097	Phenoloxidase 3	Up	/
LOC113822289	Phenoloxidase-activating enzyme	Down	/
LOC113822272	Phenoloxidase-activating factor 3	Up	Down
LOC113821695	Nuclear receptor subfamily 4 group	Down	/
LOC113813388	Nuclear factor interleukin-3	Down	/
LOC113812723	Heat shock protein	Down	Up
LOC113827906	Clotting factor B	Up	/
LOC113802295	Lysozyme C	Down	Up
LOC113805933	Lysozyme	Down	/
LOC113822201	Lysosomal-trafficking regulator	Up	/
LOC113827169	Lysosomal aspartic protease	Down	/
LOC113814483	Retinol dehydrogenase 14	Down	/
LOC113809514	Retinoic acid receptor	Down	/
LOC113828984	Serine proteinase	Up	/
LOC113808118	Early growth response protein 1	Down	/
LOC113830398	Apoptosis-inducing factor 3	Up	/
LOC113804019	Early endosome antigen 1	Up	/
LOC113820723	Histone H1.3	Up	/
LOC113825585	Cathepsin L1	Up	/

*Notes*. “/” indicated an insignificant difference.

## Data Availability

Data will be made available from the corresponding author upon reasonable request.

## Supplementary Materials

Supplementary 1 The sequence information of primer pairs used for quantitative real-time PCR.

Supplementary 2 Detailed experimental methods for hemocyte transcriptomic analysis and hemolymph metabolomics analysis.
