# Supplementary material for: Effects of Tannin Supplementation in Diet on the Resistance to Ammonia Stress of Pacific White Shrimp Litopenaeus vannamei
Source: Aquac Nutr. 2024 May 13;2024:5539701. doi: 10.1155/2024/5539701 (PMC11105962; doi:10.1155/2024/5539701)
Supplement: Supplementary 1 — The sequence information of primer pairs used for quantitative real-time PCR. [file 5539701.f1.doc]

**Supplementary 1. The sequence information of primer pairs used for quantitative real-time PCR.**

| Gene | Primer name | Sequence (5'-3') |
| --- | --- | --- |
| LOC113813658 | FORWARD | GGGAGTACCTAGTGGATGTG |
|  | REVERSE | TACCAGGATTTCCAAGTTTC |
| LOC113808907 | FORWARD | TTGAATCACTACTGGGCTAA |
|  | REVERSE | AGGAGGACGATCTTCTAAAC |
| LOC113808646 | FORWARD | AACCGCCACGAATACTACCG |
|  | REVERSE | CAATCCGCCCAATACTCCAC |
| LOC113807589 | FORWARD | CTGTGCTACTCCCACCCTCC |
|  | REVERSE | GGTAGGAAGGGTCGCACATCG |
| LOC113815968 | FORWARD | CAGAAGTTGTTGGAGGAGGC |
|  | REVERSE | AGGAGAAACGCTGACGCTAT |
| LOC113815129 | FORWARD | TGAATCCCAAGGTCAATCGC |
|  | REVERSE | AAATAGCGTGAGGAAGGGCAT |
| LOC113813432 | FORWARD | GGACGAGGAGTGCGGTGGT |
|  | REVERSE | CGAGGGTGGAACAGGTAGGG |
| LOC113806020 | FORWARD | GGCAACTGGGCGGTCAAC |
|  | REVERSE | CCTTTGCCCGACGAAGACAT |
| LOC113817069 | FORWARD | GCCAGCAGGTGTTGGGACG |
|  | REVERSE | GCTGGAGCCGCATGTACCC |
| LOC113826865 | FORWARD | ACCTGCCTACTTCTGCTTA |
|  | REVERSE | TACGATTACCCTTCACGAC |
