# Supplementary material for: Effects of Tannin Supplementation in Diet on the Resistance to Ammonia Stress of Pacific White Shrimp Litopenaeus vannamei
Source: Aquac Nutr. 2024 May 13;2024:5539701. doi: 10.1155/2024/5539701 (PMC11105962; doi:10.1155/2024/5539701)
Supplement: Supplementary 2 — Detailed experimental methods for hemocyte transcriptomic analysis and hemolymph metabolomics analysis. [file 5539701.f2.doc]

**Supplementary 2. Detailed experimental methods for hemocyte transcriptomic analysis and hemolymph metabolomics analysis.**

**1.1** **Hemocyte transcriptomic analysis**

Total RNA was extracted from the hemocyte using TRIzol® Reagent according the manufacturer’s instructions (Invitrogen), and genomic DNA was removed using DNase I (TaKara, China). Then RNA quality was determined by 2100 Bioanalyser (Agilent) and quantified using the ND-2000 (NanoDrop Technologies). A high-quality RNA sample was used to construct a sequencing library using a TruSeq™ RNA sample preparation kit from Illumina (San Diego, CA). After the mRNA library successfully passed the quality inspection, PE150 sequencing was performed using the Illumina NovaSeq 6000 platform (Thermo, Waltham, MA, USA).

After preprocessing to remove low quality reads (quality score < 30) and those containing adaptor sequences, data were assembled using the Trinity software, with transcripts > 300 bp long being retained for further analysis. To identify differential expression genes (DEGs) between different treatment of TS_0 vs TA_0, and TS_800 vs TS_0, the expression level of each transcript was calculated according to the transcripts per million reads (TPM) method. RSEM (http://deweylab.biostat.wisc.edu/rsem/) was used to quantify gene abundances. Essentially, differential expression analysis was performed using the Cuffdiff (http://cufflinks.cbcb.umd.edu/). In addition, functional-enrichment analysis including GO and Kyoto encyclopedia of genes and genomes (KEGG) were performed to identify which DEGs were significantly enriched in GO terms and metabolic pathways at Bonferroni-corrected *P*-value ≤ 0.05 compared with the whole-transcriptome background. GO functional enrichment and KEGG pathway analysis were carried out by Goatools (https://github.com/tanghaibao/Goatools) and KOBAS (http://kobas.cbi.pku.edu.cn/home.do). The statistical analyses of the GO and KEGG enrichment were set as Bonferroni-corrected *P*-values < 0.05.

**1.2** **Hemolymph metabolomics analysis**

Hemolymph samples (n = 6) from the four treatments were used for metabolomic analysis. All the hemolymph samples were taken from liquid nitrogen and thawed at 4 °C, and metabolite extraction was dissolved with 80% methanol solution. Twenty microliters of each sample were taken to quality control (QC) samples, and the rest of the samples was used for LC-MS detection. The metabolites were analyzed using a gas chromatograph system coupled to an Thermo Vanquish system equipped with an ACQUITY UPLC® HSS T3 (150×2.1 mm, 1.8 μm, Waters) column. Mass spectrometry was executed on a Thermo Q Exactive mass spectrometer. Data-dependent acquisition (DDA) MS/MS experiments were performed with HCD scans. Dynamic exclusion was implemented to remove some unnecessary information in the MS/MS spectra.

Base peak chromatograms (BPCs) were obtained through continuous description of the ions with the highest intensity in each mass spectrogram. All the data were determined using quality control (QC) and quality assurance (QA). Multivariate analyses including principal component analysis (PCA) and orthogonal partial least squares discrimination analysis (PLS-DA) were conducted by using the SIMCA 14.1 software package (V14.1, MKS Data Analytics Solutions, Umea, Sweden). All the metabolites were classified according to KEGG and Metabolon.inc. Based on the exact mass match (error < 15 ppm) and secondary spectra MS/MS, metabolite identification was performed by searching Metlin (http://metlin.scripps.edu/), MoNA (https://mona. fiehnlab.ucdavis.edu/) and company databases (BioNovoGene, China). Differential metabolites (DMs) of TS_0 vs TA_0 and TS_800 vs TS_0 was identified. The PLS-DA model was validated using a permutation test with 200 as the permutation number. Student’s t-test (*P* < 0.05) combined with the first principal component of variable importance in projection (VIP) values (VIP ≥ 1) were used to determine the species distribution models (SDMs) among the pairwise comparison groups. Agglomerate hierarchical clustering of the DMs was performed using the R software (v3.3.2) pheatmap package. The DMs were annotated with KEGG pathway analysis using metaboanalyst software (www. metaboanalyst.ca).
